# Control of lipid metabolism by adipocyte FGFR1-mediated adipohepatic communication during hepatic stress

**DOI:** 10.1186/1743-7075-9-94

**Published:** 2012-10-30

**Authors:** Chaofeng Yang, Cong Wang, Min Ye, Chengliu Jin, Weimin He, Fen Wang, Wallace L McKeehan, Yongde Luo

**Affiliations:** 1 Center for Cancer and Stem Cell Biology, Institute of Biosciences and Technology, Texas A&M Health Science Center, 2121 W. Holcombe Blvd, Houston, TX, 77030-3303, USA; 2 IBT Proteomics and Nanotechnology Laboratory, Institute of Biosciences and Technology, Texas A&M Health Science Center, 2121 W. Holcombe Blvd, Houston, TX, 77030-3303, USA; 3 Center for Environmental and Genetic Medicine, Institute of Biosciences and Technology, Texas A&M Health Science Center, 2121 W. Holcombe Blvd, Houston, TX, 77030-3303, USA; 4 Department of Animal Resources, Georgia State University, Atlanta, GA, 30303, USA; 5 Center for Cardiovascular Diseases, Texas Southern University, Houston, TX, 77004, USA; 6 School of Pharmaceutical Science, Wenzhou Medical College, Wenzhou, 325035, China

**Keywords:** Adipose tissue, Fibroblast growth factor, FGF, Fibroblast growth factor receptor, FGFR, Hepatic steatosis, Hepatic stress, Lipid metabolism, Starvation

## Abstract

**Background:**

Endocrine FGF19 and FGF21 exert their effects on metabolic homeostasis through fibroblast growth factor receptor (FGFR) and co-factor betaKlotho (KLB). Ileal FGF19 regulates bile acid metabolism through specifically FGFR4-KLB in hepatocytes where FGFR1 is not significant. Both FGF19 and FGF21 activate FGFR1-KLB whose function predominates in adipocytes. Recent studies using administration of FGF19 and FGF21 and genetic ablation of KLB or adipocyte FGFR1 indicate that FGFR1-KLB mediates the response of adipocytes to both FGF21 and FGF19. Here we show that adipose FGFR1 regulates lipid metabolism through direct effect on adipose tissue and indirect effects on liver under starvation conditions that cause hepatic stress.

**Methods:**

We employed adipocyte-specific ablations of FGFR1 and FGFR2 genes in mice, and analyzed metabolic consequences in adipose tissue, liver and systemic parameters under normal, fasting and starvation conditions.

**Results:**

Under normal conditions, the ablation of adipose FGFR1 had little effect on adipocytes, but caused shifts in expression of hepatic genes involved in lipid metabolism. Starvation conditions precipitated a concurrent elevation of serum triglycerides and non-esterified fatty acids, and increased hepatic steatosis and adipose lipolysis in the FGFR1-deficient mice. Little effect on glucose or ketone bodies due to the FGFR1 deficiency was observed.

**Conclusions:**

Our results suggest an adipocyte-hepatocyte communication network mediated by adipocyte FGFR1 that concurrently dampens hepatic lipogenesis and adipocyte lipolysis. We propose that this serves overall to mete out and extend lipid reserves for neural fuels (glucose and ketone bodies), while at the same time governing extent of hepatosteatosis during metabolic extremes and other conditions causing hepatic stress.

## Background

FGF19 (FGF15 in mice) and FGF21 are circulating endocrine factors that affect metabolism and metabolic diseases including obesity and diabetes [1-4]. FGF19 fluctuates with normal feeding-fasting cycles and is induced in the ileum by postprandial bile acids and ligands of the farnesoid X receptor (FXR) [5-7]. In contrast, FGF21 is induced in the liver in response to liver perturbation [8-11] and during metabolic extremes that include those induced by starvation and obesity [12-14]. Normally hepatic expression and blood level of FGF21 are low and vary widely among individuals, but become consistently elevated during starvation [13].

Genetic deletion experiments have revealed that FGF19 regulates hepatic cholesterol/bile acid synthesis specifically through hepatic FGFR4 in partnership with transmembrane co-receptor KLB [6,15,16]. The direct tissue target and FGFR isotype that mediate FGF21 action and metabolic effects of FGF19 in addition to hepatic bile acid metabolism are unclear. Recently we showed that in addition to FGFR4-KLB, FGF19 also binds with high affinity and activates FGFR1-KLB [17,18]. In contrast, FGF21 binds with high affinity and activates only FGFR1-KLB. FGFR4 is the predominant FGFR isotype expressed in hepatocytes whereas FGFR1 is expressed at low to negligible levels [18-20]. FGFR1 is expressed along with KLB prominently in adipocytes where other FGFRs including FGFR4 are very low or absent [18,19]. In view of the FGFR-KLB binding profile for FGF19 and FGF21, FGFR1 in adipocytes is a candidate target for FGF21 specifically, while both FGFR1 in adipocytes and FGFR4 in hepatocytes are targets for FGF19. Based on response of signal transduction indicators downstream of FGFR, adipose tissue specifically responds to infusions of FGF21 relative to liver while both tissues are responsive to FGF19. The responses in adipocytes were abrogated in mice deficient in adipose FGFR1 or KLB [18].

Manipulation of expression or pharmacological administration of FGF19 or FGF21 protein in mice has major impact on metabolic diseases and pathways in hepatocytes in addition to adipocytes [14,21-24]. A collaborative investigation in parallel to this study with our mouse model deficient in adipose FGFR1 reveals that, under conditions of diet-induced obesity (DIO) and FGF21 administration, adipose tissue expressing FGFR1-KLB accounts for nearly the entirety of beneficial metabolic effects of FGF21 *in vivo*^a^. Under dietary restriction such as fasting and starvation conditions, FGF21 was proposed as a starvation hormone and a paracrine factor acting on liver [13,14]. However, it is unclear whether the observed metabolic effects in liver under these conditions relative to normal fed state in these two tissues are a direct consequence of FGF-activated FGFR-KLB signaling in the respective tissue, or an indirect consequence in the other through systemic inter-organ communication. In this report, we analyzed under normal and starvation conditions the impact of adipocyte-specific deletion of FGFR1 on metabolic parameters associated with adipose tissue and liver. The deletion caused little change in adipocyte metabolic gene expression at the transcription level, but instead caused elevation in expression of predominantly lipogenic genes in the liver. The effects of the adipocyte FGFR1 deficit on hepatic lipid metabolism were particularly evident under the metabolic extreme of starvation. From the observations, we conclude that liver is a major indirect response organ to FGFR1 signaling in adipocytes. Adipocyte FGFR1 serves to underpin a systemic communication between hepatocytes and adipocytes mediated by FGF21 production from hepatocytes under stress that signals through adipose FGFR1. Flux of free fatty acids and adipokines from adipocytes regulated by FGFR1 in turn communicates back to liver to limit hepatic lipogenic gene expression and extent of hepatic steatosis and associated stress. Under these conditions, FGFR1 concurrently dampened lipolytic activity in the adipocytes. We propose an axis of hepatocyte-adipocyte cooperation mediated by hepatocyte FGF21 and adipocyte FGFR1 that serves to protect and mete out lipid reserves systemically while protecting the liver against excessive steatosis and damage under metabolic extremes and general hepatic stress.

## Materials and methods

### Animals

The *FGFR1*-floxed (FGFR1^lox/lox^, referred to hereafter as FGFR1Fx or control), *FGFR2*-floxed (FGFR2Fx) and aP2-Cre mice have been described [25-27]. These mice were backcrossed to C57BL/J6 background for more than 5 generations. Mice with deficiency in FGFR1 and FGFR2 specifically in adipocytes were generated by crossing the FGFR1Fx and FGFR2Fx mice with aP2-Cre mice as described (FGFR1^lox/lox^aP2^Cre^ and FGFR2^lox/lox^aP2^Cre^, referred to hereafter as FGFR1Cn and FGFR2Cn), respectively [18]. Experimental animals were male. The 12 h fast began at 6:00 PM with 70 percent of the time duration in dark cycle when murine daily food intake occurs. The 48 h fast began at 6:00 AM when the light cycle started. All mice were housed in the Program of Animal Resources in the Institute of Biosciences and Technology, and were handled in accordance with the *principles and procedure of the Guide for the Care and Use of Laboratory Animals*. All experimental procedures were approved by the Institute of Biosciences and Technology Institutional Animal Care and Use Committee (IBT IACUC) with protocol #10022 entitled “BetaKlotho-FGFR in the liver” and #09008 entitled “IBT-Mouse Models”.

### Analysis of gene expression

Total RNA isolation and quantitative PCR were done as described [18]. Representative liver tissues were from the left lobe, and gonadal adipose tissues were used as representative for the adipose tissue, from the number of mice as indicated.

### Cellular fractionation of adipose tissue

Adipocytes were separated from the stromal-vascular (SV) fraction by a modified Rodbell method [28]. Adenosine was added to suppress lipolysis. About 2 gram of fat from male mice at ages of eight to ten weeks were minced into 2–3 mm diameter pieces and dissociated with 3 mg/ml collagenase in Krebs–Ringer-Hepes (KRH) buffer [129 mM NaCl, 5 mM NaHCO_3_, 4.8 mM KCl, 1.2 mM KH_2_PO_4_, 1 mM CaCl_2_, 1.2 mM MgCl_2_, 2.8 mM glucose and 10 mM Hepes (pH7.4)], at 37°C with slow shaking for 1 h. After filtration through 250 μm gauze meshes, the adipocyte and SV fractions were separated by centrifugation at 200 g for 5 min. Adipocytes were carefully harvested from the top fat cake layer and the SV fraction was from the pellet on the bottom.

### Liver and adipose tissue histology

Portions of freshly isolated liver and adipose tissues were fixed with Histochoice Tissue Fixative MB (Amresco, Solon, OH) and paraffin-embedded. Tissue sections were stained for general pathological examination with hematoxylin and eosin. In addition, liver tissue was processed in Neg-50 frozen section medium. Lipid droplets were revealed by staining the frozen section with Oil Red O at 60°C for 8 min. After washing with 85% isopropanol, the sections were further counterstained with hematoxylin.

### Serum metabolic parameters and liver lipids

Blood was collected by retro-orbital puncture after anesthetization with 2,2,2-tribromoethanol (avertin). Serum was prepared by centrifugation at 2000 *g* and stored in aliquots at −80°C. Lipids were extracted from homogenates of 50 mg tissue with 1 ml chloroform/methanol (2:1) overnight. After centrifugation at 12000 *g* for 15 min, lipids in the lower organic phase were collected and evaporated in a rotary speed vacuum. Lipid pellets were dissolved in PBS containing 1% Triton X-100. Serum parameters were measured with standard assay kits including triglyceride (TG) (Infinity, Middletown, VA), NEFA and cholesterol (Wako Pure Chemicals, Richmond, VA), and ketone bodies (Stanbio, Boerne, TX). Serum insulin and glucose levels were determined by a Glucometer (Bayer, Elkhart, IN).

### Lipase activity

Gonadal adipose tissue was homogenized in cold lysis buffer (0.25 M sucrose, 10 mM Tris–HCl, 1 M NaCl, 2 mM EDTA, pH 7.4). Lysate was centrifuged at 10000 x g for 30 min and the clear aqueous phase was transferred for analysis. The rate of lipoprotein lipase (LPL) activity per unit extract protein that hydrolyzes triglycerides associated with VLDL was measured by an assay kit (Cat # RB-LPL2, Roar biomedical, Inc., Calverton, NY) according to the manufacturer’s instructions and rates determined from the slope of time curves between 20 and 40 min.

### Adipokine array

Equal amounts of serum samples from FGFR1Cn or FGFR1Fx mice under different diet restriction conditions as indicated, were used to perform antibody array analysis against selected adipokines following the manufacturer’s protocol (R&D Systems, Minneapolis, MN).

### Serum activities of liver enzymes ALT and AST

The blood samples from FGFR1Fx and FGFR1Cn mice (n=6 each group) were collected after fed or fasted for 48 h. The activities of liver blood enzymes alanine aminotransferase (ALT) and aspartate aminotransferase (AST) were measured with an enzymatic activity assay kit and human based serum calibrator (DC-CAL) (Sekisui diagnostics, Charlottetown, PE Canada).

### Statistical analysis

Experiments were reproduced three times independently with triplicates for each experiment. A representative of three or more experiments may be shown in micrographs. Where indicated, the mean and standard deviation (SD) was determined. Comparisons between different genotype groups were performed with the unpaired *t* test. Values were deemed to be statistically significantly different at p≤0.05.

## Results

### Ablation of FGFR1 in adipocytes causes changes in hepatic, but not adipocyte metabolic gene expression

Mice deficient in FGFR1 in adipocytes (FGFR1^lox/lox^aP2^Cre^ or FGFR1Cn) were generated from mice with the floxed *FGFR1* gene (FGFR1^lox/lox^ or FGFR1Fx) [26] and mice expressing Cre recombinase driven by the promoter of adipocyte fatty acid binding protein aP2 [25,29]. LacZ staining in mice with both aP2-Cre+: ROSA26R+ alleles [27] as compared to the control indicated that the aP2 promoter was active in a majority of adipocytes (Additional file 1: Figure S1A and B). When compared to liver, muscle and kidney, the expression of FGFR1 in total adipose tissue of the FGFR1Cn mice was reduced by 50 percent (Additional file 1: Figure S1C). To determine efficacy of the FGFR1 ablation specifically in the adipocyte fraction, adipose tissue was fractionated into mature adipocyte and the stromal-vascular (SV) fractions based on aP2 expression. FGFR1 mRNA in the adipocyte fraction of the FGFR1Cn mice was reduced to less than 5 percent that of wildtype or FGFR1^lox/lox^ control mice and to 50 percent in the stromal-vascular fraction (Additional file 1: Figure S1D). Expression of FGFR2 in mature adipocytes was unchanged in the FGFR1Cn mice. Expression of co-factor KLB in mature adipocytes was over 15 times that in the stromal-vascular fraction and was unaffected by the absence of FGFR1.

**Figure 1:**
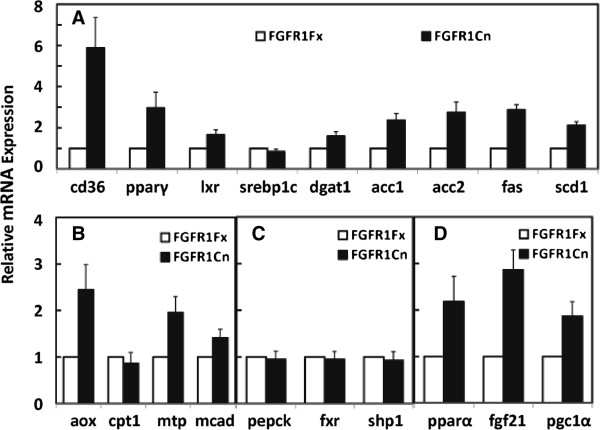
**Effects of adipocyte FGFR1 deficiency on hepatic metabolic gene expression during normal feeding-fasting.** Relative expression of the indicated genes in the liver (Additional file 1: Table S1) involved in lipogenesis (**A**), fatty acid transport and oxidation (**B**), gluconeogenesis and bile acid/cholesterol metabolism (**C**) and FGF21 transcriptional regulation (**D**), were assessed by quantitative PCR in control and adipocyte-deficient FGFR1 mice in normal fed state or after a 4 h fast. Expression in control FGFR1Fx mice was assigned a value of 1. Data are the mean ± SD of 10 mice with p<0.05 for all tests.

The absence of FGFR1 in the adipocytes precipitated significant changes at the mRNA level in expression of hepatic genes involved in lipid metabolism. The shifts predominantly were relative increases in expression of those associated with lipogenesis (Figure 1A). The mRNA for membrane fatty acid transporter CD36 was increased by a dramatic 6 fold accompanied by an increase of 3 fold in the key lipogenic transcriptional regulator PPARγ. Acyl-CoA oxidase (AOX) expression, an enzyme involved in polyunsaturated fatty acid genesis and degradation of very long chain FFA, was elevated by 2.5 fold. However, expression of two genes coding for factors associated with fatty acid oxidation, MTP and MCAD, was also increased (Figure 1B). No changes were observed in genes associated with cholesterol/bile acid metabolism or gluconeogenesis (Figure 1C). Notably, a 3 fold increase in hepatic and systemic FGF21 expression was observed (Figure 1D, Additional file 1: Figure S2). This was coincident with significant increases in expression of reported regulators for hepatic FGF21 expression, PPARα and PGC1α [14,23] in response to absence of adipocyte FGFR1 (Figure 1D).

**Figure 2:**
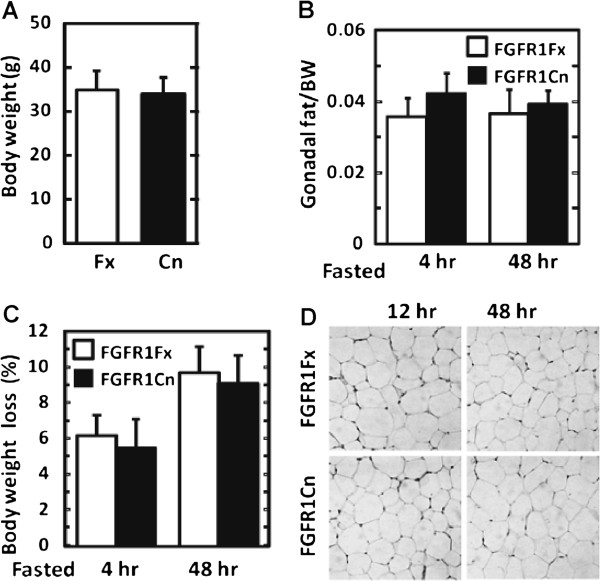
**Lack of overt abnormalities in mice lacking adipocyte FGFR1.** (**A**) Change in total body weight (BW) between FGFR1Fx and FGFR1Cn mice. (**B**) Change in ratio of gonadal fat to body weight after 4 h fasting and 48 h starvation. (**C**) Change in body weight loss. (**D**) Change in white adipose tissue morphology during 12 and 48 h starved states. Data are the mean ± SD (n=10), p<0.05 for all tests.

These adipocyte FGFR1-dependent shifts in hepatic metabolic gene expression occurred in the absence of overt changes in body weight, adiposity and morphology of adipocytes or hepatocytes (Figure 2A-2D), in serum metabolic parameters including triglycerides, NEFA, glucose and ketone bodies (Additional file 1: Figure S3) and insulin (not showed), and in expression at the mRNA level of a variety of genes in adipose tissue that code for metabolic parameters (Additional file 1: Figure S4). This indicates that in normal physiology, adipocyte FGFR1 underlies systemic signals from adipocytes to hepatocytes that impact hepatic metabolic gene expression that is in largest part depression of those involved in lipogenesis.

### The absence of adipocyte FGFR1 results in increase of starvation-induced hepatic steatosis coincident with increases in hepatic lipogenic gene expression

**Figure 3:**
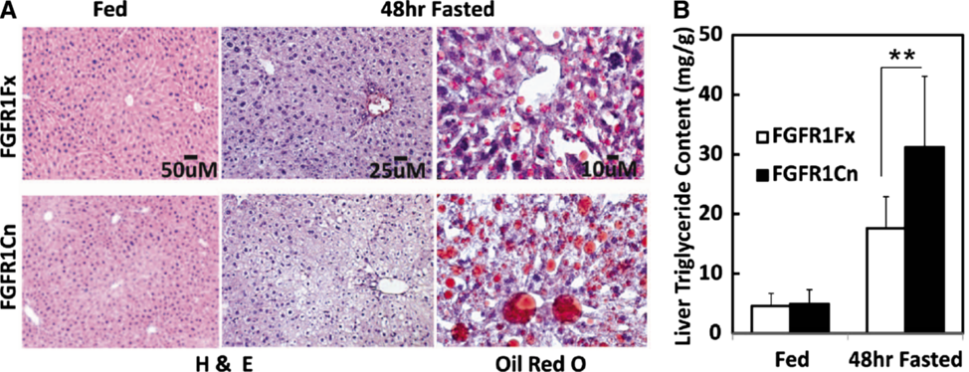
**Increased starvation-induced fatty liver and lipid content in mice deficient in adipose FGFR1.** (**A**) Appearance of liver tissue. Conventional hematoxylin and eosin (H&E) staining on liver sections revealed the elevated characteristic vacuoles induced by lipid accumulation. Staining by Oil Red O revealed an increase in the number and size of lipid droplets. (**B**) Quantitation of liver TG content. Data are the mean + SD (n=7-8). **p<0.005, and p<0.05 for all other tests.

**Figure 4:**
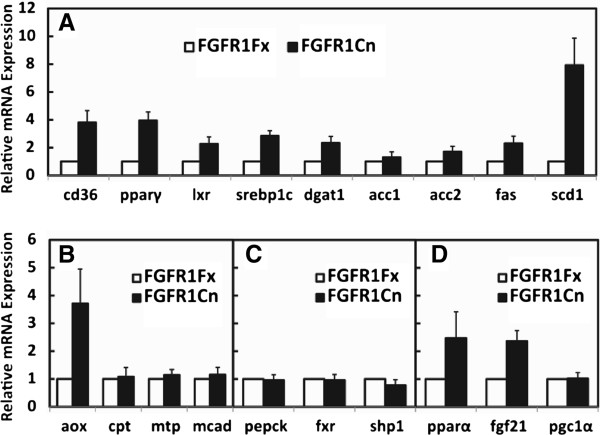
**Increase in gene expression of hepatic lipogenic enzymes caused by absence of adipose FGFR1 after starvation.** Expression of the indicated hepatic genes as described in Figure 1 (Additional file 1: Table S1) was assessed by quantitative PCR in control and adipocyte-deficient FGFR1 mice after food restriction for 48 h. Expression level in FGFR1Fx mice was assigned a value of 1. Data are the mean ± SD (n= 10), p<0.05 for all tests.

No changes in hepatic lipid content or overt hepatocyte morphology were observed due to the absence of adipocyte FGFR1 under normal feeding conditions. Starvation is an extreme metabolic condition that imposes stress on the liver and is evident by development of hepatic steatosis. Under starvation condition, candidate activating endocrine ligands, FGF21 [13,14] and FGF19 [5,7], are also induced to sustained elevated levels. Therefore, we imposed a prolonged 48 h starvation on mice to test for additional phenotypes in liver, adipose tissue and serum. The deficit of adipocyte FGFR1 increased the severity of the hepatic steatosis (Figure 3A). A 1.5 fold increase in hepatic triglyceride (TG) content coincident (Figure 3B) with elevation of expression of hepatic genes specifically involved in lipid metabolism, not glucose or ketone body metabolism, was also evident in the deficient mice. The changes in hepatic gene expression due to absence of adipocyte FGFR1 are still tightly clustered around lipogenic genes similar to observations during normal feeding conditions (Figure 4). Notable differences caused by the adipocyte FGFR1 deficiency between normal feeding and the 48 h starvation conditions were elevations of SREBP1c and SCD1 by 2.5 and 6 fold (Figure 4A), respectively, and lack of increases in fatty acid degradative factors MTP and MCAD (Figure 4B) and in regulators for bile acids metabolism and gluconeogenesis (Figure 4C). The increase of SREBP1c, a fatty liver and hepatic ER stress marker [30], indicates that an increase of hepatic stress may accompany the exaggerated lipogenesis and steatosis caused by the metabolic stress imposed by starvation and adipose FGFR1 deficit. Prolonged starvation significantly increases hepatic expression of Nrf2 and Ucp2 but not MnSOD, which are oxidative stress markers; however, an effect of the adipose FGFR1 deficit was not statistically significant (Additional file 1: Figure S5). A lack of increases in MTP and MCAD in the liver under prolonged starvation is also consistent with the increase of hepatic lipogenesis and steatosis in the adipose FGFR1 deficient mice. The elevation of PPARα and hepatic FGF21 mRNAs caused by the loss of adipocyte FGFR1 observed during normal feeding remained intact in the starvation conditions, while the effect on PGC1α expression and systemic FGF21 was masked or at a maximal level (Figure 4D, Additional file 1: Figure S2). These results suggest that when liver is under metabolic stress induced by starvation and steatotic conditions, adipocyte FGFR1 and its activating ligands underlie signals from adipocytes to hepatocytes that overall dampen hepatic lipogenic gene expression and limit extent of starvation-induced hepatic steatosis.

### Serum NEFA and triglycerides, but not glucose or ketone bodies, are concurrently elevated in starved mice deficient in adipocyte FGFR1

**Figure 5:**
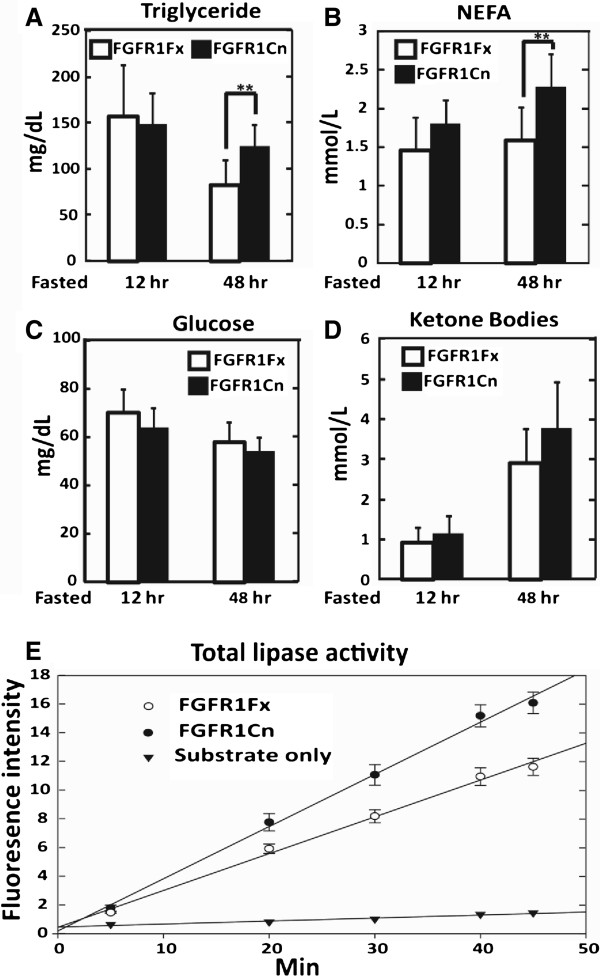
**Elevated serum TG, NEFA and adipose lipase activity induced by starvation stress in mice deficient in adipocyte FGFR1.** (**A**) Serum triglycerides, (**B**) NEFA, (**C**) glucose and (**D**) ketone bodies were analyzed in FGFR1Cn and FGFR1Fx mice after food restriction for 12 and 48 h. Data are the mean ± SD (n = 10), ** p<0.005, and p<0.05 for all other tests. (**E**) Adipocyte total lipase activity. Rate of lipase activity was assessed by fluorescence as described in Materials and Methods in mice starved for 48 h. Data are the mean ± SD (n = 6).

As noted earlier despite shifts in primarily hepatic lipogenic gene expression including systemic FGF21, no changes in serum NEFA, triglycerides, glucose or ketone bodies during normal feeding and even after a 24 h fast could be clearly detected as a consequence of the adipocyte FGFR1 deficiency. However, imposition of the stress of starvation conditions when hepatic steatosis is apparent caused a detectable rise in serum TG and NEFA levels at 1.5 and 1.4 fold that of wildtype or FGFR1Fx mice (Figure 5A and B). In contrast, neither the depressed glucose levels (Figure 5C) nor elevated ketone bodies elicited by starvation (Figure 5D) changed between control and deficient mice. These starvation-dependent observations on serum parameters elicited by the adipocyte FGFR1 deficiency are consistent with the general elevation of specifically hepatic lipogenic genes and hepatic steatosis, without effect on hepatic genes involved in glucose and ketone body metabolism. The forced increase in NEFA level from adipocytes due to FGFR1 deficit under prolonged starvation, likely in part resulted in the observed compensatory increases of hepatic and serum FGF21 through hepatic PPARα, and the elevated hepatic lipogenesis and steatosis as well.

To further quantitatively determine the hepatic stress level resulting from exaggerated hepatic steatosis due to the adipose FGFR1 deficiency, we analyzed blood activities of the two liver enzymes alanine aminotransferase (ALT) and aspartate aminotransferase (AST). Serum ALT and AST are indicators for pathological changes in hepatocytes and liver damage/injury and diseases. There is no significant change in serum activities of ALT and AST upon adipose FGFR1 deficiency in the normal fed state. Starvation increases the activities of serum ALT at about 8% and 22% and AST at about 49% and 80% in the FGFR1Fx and FGFR1Cn mice, respectively. These results demonstrate that adipose FGFR1 deficiency significantly elevates the serum enzyme activities of both ALT and AST, indicating significant exaggeration of liver stress levels upon adipose FGFR1 deficiency in starvation (Additional file 1: Figure S6).

### The adipocyte FGFR1 deficit causes elevated adipocyte lipase activity during starvation

To determine whether adipocytes participate directly in the starvation-dependent phenotypic effects of the adipocyte FGFR1 deficiency, we again tested for changes in adipocyte gene expression. Regulation of expression of adiponectin by adipose FGFR1 signaling has been demonstrated ^a^. However, little effect of the adipocyte FGFR1 deficit on expression of many other metabolic genes coding for major metabolic enzymes and regulators at the mRNA level, including hsl and atgl, in adipose tissue was observed, similar to normal feeding and short-term fasting (Additional file 1: Figure S4). Notably, the rate of lipoprotein lipase enzyme activity in adipocyte extracts from the FGFR1Cn mice was about 1.45 times that of control mice (Figure 5E). The change in adipose lipase enzyme activity but no effect at the mRNA level suggested a post-transcriptional regulation of lipase activity by adipocyte FGFR1 signaling. This result indicates that under starvation conditions, when an activating FGF ligand is sufficient, adipocyte FGFR1 serves to dampen lipolysis in the adipocytes while concurrently eliciting signals that dampen lipogenesis and steatosis in hepatocytes.

### Ablation of adipose FGFR2 does not elicit the hepatic response elicited by the FGFR1 deficiency

**Figure 6:**
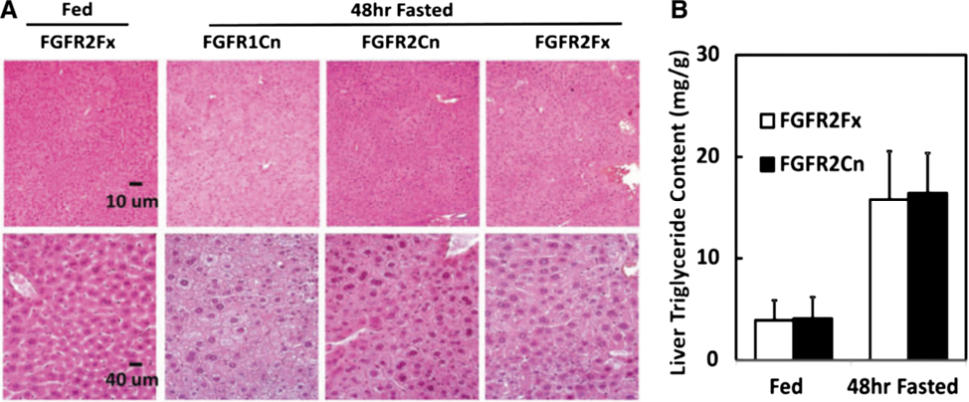
**FGFR2 deficiency in adipocytes exerted no comparable effect on starvation-induced fatty liver.** (**A**) Histology of liver tissue from adipocyte-deficient FGFR2 (FGFR2Cn) mice was compared to that from the FGFR1Cn and FGFR2Fx mice in both fed and 48 h starvation states at two magnifications. The enhanced steatotic vacuolization observed in FGFR1Cn mice was not apparent in the FGFR2Cn mouse livers. (**B**) Quantitation of liver TG content from FGFR2Fx and FGFR2Cn mice. Data are the mean ± SD (n= 10), p<0.05 for all other tests.

FGFR2 is expressed at the mRNA level in adipocytes and adipose tissue (Additional file 1: Figure S1D). To address whether FGFR2 played a similar role to FGFR1 in adipose tissue, we prepared a mouse line deficient in adipocyte (FGFR2 FGFR2^lox/lox^aP2^Cre^ or FGFR2Cn) following the same approach as described for FGFR1. In agreement with a previous report [31], the FGFR2 deficient mice exhibited no overt abnormalities in development and cellular homeostasis. Moreover, the elevation in serum TG content and starvation-induced hepatic steatosis caused by the adipocyte FGFR1 deficiency was not observed in animals deficient in adipocyte FGFR2 (Figure 6A and B). This indicates that adipocyte FGFR1, but not FGFR2 underpins the phenotypes in lipid metabolic homeostasis observed in this study.

## Discussion

Here we deduce from results of gene ablation, that under normal dietary condition, FGFR1 in adipocytes normally underlies signals from adipocytes that restrict hepatic expression levels of a subset of genes involved in lipid metabolism. The subset is in largest part genes associated with hepatic lipogenesis. This occurs without apparent changes in adipocyte metabolic gene expression, expression of hepatic genes involved in glucose metabolism, blood levels of glucose and lipids and morphology of adipocytes and hepatocytes.

In the presence of transmembrane co-receptor KLB, both FGF19 and FGF21 bind with high affinity and activate FGFR1 [17,18,20]. In contrast to FGF19, FGF21 fails to bind and activate FGFR4-KLB that is the predominant FGFR in hepatocytes. FGF21 is specific for the FGFR1-KLB complex which is the predominant FGFR isotype in adipose tissue [18,20,32]. This differential specificity limits the direct action of FGF21 to adipose tissue relative to liver, in contrast to the activity of FGF19 which acts potentially on both adipocytes via FGFR1 and hepatocytes via FGFR4. The activation of adipose tissue by FGF21 through FGFR1 was suggested by genetic deletion of FGFR1 in adipocytes in mice used in the current study [18], and this tissue- and molecule-specific actions of FGF21 underlies the breadth of its *in vivo* beneficial effects on treatment of obesity and diabetes ^a^. FGF19 is a diurnal hormone that fluctuates during normal feeding [7]. FGF21 comes into play after prolonged periods of caloric restriction or hepatic perturbation by conditions such as steatosis or chemical damage [8-14]. This suggests that during normal feeding, ileal FGF19 is likely the activating endocrine FGF for adipocyte FGFR1. Thus abrogation of FGF19-adipocyte FGFR1 signaling may underlie effects of the FGFR1 deficiency on hepatic lipid gene expression under normal conditions. Such indirect effects of FGF19 on hepatic lipogenic gene expression through adipocyte FGFR1-KLB are in addition to the direct effects on hepatic bile acid [6,15,16] and lipid metabolism mediated by FGFR4-KLB [9]. The impact of the changes in hepatic gene expression contributed by adipocyte FGF19-FGFR1 signaling during normal feeding on the hepatic contribution to overall metabolic homeostasis remains to be determined. Notably, the deficiency of FGFR1 in adipocytes also caused an increase in hepatic FGF21 without apparent effect on ileal FGF15 (Yang C, unpublished data). This indicates that during normal feeding FGF19 working through adipocyte FGFR1 may suppress expression of hepatic FGF21. This may be the reason why normally FGF21 levels are low and variable, and only rise to high sustained levels during severe metabolic extreme as starvation and other causes of hepatic stress or perturbation.

**Figure 7:**
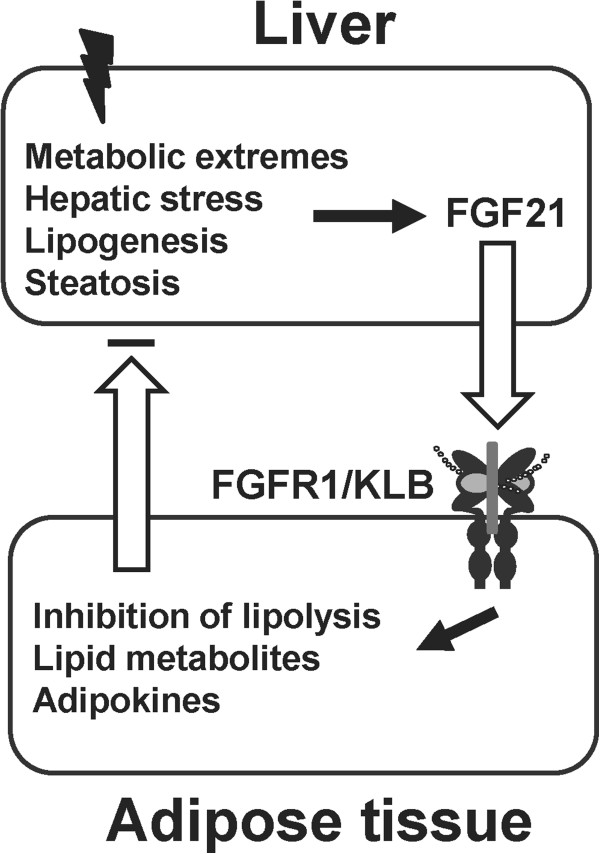
**An adipocyte FGFR1-mediated hepatocyte-adipocyte communication axis during hepatic stress.** Diverse conditions as the metabolic extremes of starvation, obesity and chemical and biological insults induce FGF21 that signals the stress to adipocytes. FGF21 activates adipose FGFR1 signaling to inhibit adipocyte lipolysis and to activate signals back to the liver that collectively dampen lipogenesis, steatosis and hepatic stress.

We used starvation conditions to impose metabolic stress on the liver that is evident by overt hepatic steatosis. Under these conditions, we observed systemic and hepatic metabolic consequences due to the adipocyte FGFR1 deficiency. We therefore deduce that under conditions as starvation that causes severe hepatic stress, the role of adipocyte FGFR1 and its cognate activating ligands underlies signals that restrict adipocyte lipolysis and through adipocyte to hepatocyte signals concurrently restrict extent of hepatic lipogenesis. These signals likely include free fatty acids and/or adipokines such as adiponectin, as a result of changes in lipolysis and endocrine function of adipose tissue governed by adipose FGFR1 signaling. Increased free fatty acids from adipose tissue deficient in FGFR1 further enhance the expression of hepatic FGF21 through its nuclear receptor PPARα, whose expression is also increased under these conditions that may be due to change in adiponectin level [33,34]^a^. Increased free fatty acids also contribute to exacerbated hepatic steatosis. These results suggest an inter-organ communication between adipose tissue and the liver mediated by hepatic FGF21 and adipose FGFR1 through adipocyte signals that include free fatty acids and/or adipokines. In view of the absence of overt phenotypic effects during normal feeding beyond the changes in hepatic lipid gene expression, our results suggest that adipocyte FGFR1 plays its most important physiological role under metabolic extremes and other conditions that stress the liver. Such conditions induce sustained and maximal levels of serum FGF21 [13,14] and FGF19 [5], and thus are conditions where endocrine activation of adipocyte FGFR1 would be maximal. We propose that the phenotypes observed using starvation as a specific metabolic extreme may reflect a general hepatic-adipocyte communication network, which is comprised of hepatic FGF21 induced by general metabolic or other type of stress on the liver, adipocyte FGFR1 and FGFR1-mediated metabolite and adipokine signals such as free fatty acids and adiponectin or other yet to be identified factors back to the liver (Figure 7). In addition to starvation, an increase in hepatic FGF21 generally accompanies hepatic stress-inducing conditions of obesity and chemical insult, infection and inflammation [8-14,35,36]. This communication primarily governed by adipose FGFR1 serves to limit adipose lipolysis and hepatic steatosis, liver stress and resultant damage under stressful or adverse conditions.

A hallmark of the starvation-induced signaling axis from hepatic FGF21 to adipocyte FGFR1 indicated by our study, is the concurrent changes in adipocyte lipolysis and hepatic lipogenesis partitioned between the two organs. Under the starvation conditions, the changes in lipid metabolism also appear relatively independent of glucose and ketone body metabolism. Normally lipid metabolism including both lipolysis and lipogenesis is tightly coupled to glucose and ketone body metabolism in overall metabolic homeostasis. Moreover, overall lipolysis and lipogenesis are tightly coupled and inversely related to each other. During the course of starvation, glucose levels progressively sink. The uncoupling promoted by the hepatocyte-adipocyte axis may serve to slow down the flow of lipids into glucose and ketone bodies. This extends critical lipid stores for as long as possible for maintenance of brain fuels above critically low levels until feeding resumes. This metabolic uncoupling promoted by the hepatic FGF21-adipocyte FGFR1 axis may be of temporary benefit to the organism under conditions of not only starvation, but also a variety of other conditions that are stressful to the liver when overloaded beyond its routine role in metabolic homeostasis. Reversible hepatic steatosis generally accompanies such conditions. The endocrine FGF-mediated hepatocyte-adipocyte axis may also serve to limit hepatic steatosis before it becomes irreversibly damaging.

## Conclusions

Adipocyte-specific deletion of FGFR isotypes, FGFR1 and FGFR2 show that specifically adipose FGFR1 mediates indirect effects in liver that are most significant under starvation conditions which causes hepatic stress and steatosis. This occurs through direct adipose FGFR1-dependent restrictions on adipocyte lipolysis [37] and indirectly hepatic lipogenesis through systemic adipocyte to hepatocyte signals. This communication also serves to attenuate extent of compensatory hepatic steatosis that often occurs during hepatic stress. This adipohepatic communication cycle also serves overall to mete out and extend lipid reserves for neural fuels (glucose and ketone bodies) during metabolic extremes and other conditions causing hepatic stress. This is particularly of benefit, possibly life-saving, during prolonged starvation to preserve consciousness until feeding opportunity occurs. Since adipocyte FGFR1 is a selective target of FGF21 and an additional target of FGF19 in addition to hepatocyte FGFR4, our results predict that this adipocyte-directed mechanism [38,39] may underpin the beneficial effects of endocrine ligands, FGF21 and FGF19, observed under conditions of not only caloric restriction, but also excess as in obesity that both cause metabolic perturbation in the liver.

## Endnotes

^a^Accepted manuscript: Andrew C. Adams, Chaofeng Yang, Tamer Coskun, Christine C. Cheng, Ruth E. Gimeno, Yongde Luo, Alexei Kharitonenkov. The breadth of FGF21’s metabolic actions are governed by FGFR1 in adipose tissue. Molecular Metabolism. [posted online 17 August, 2012].

## Competing interests

The authors declare that they have no competing interests.

## Authors' contributions

CY, WLM and YL designed research. CY, MY, CJ and YL performed research. WH and FW contributed reagents. CY, YL and WLM analyzed data. CY, WLM and YL wrote the paper. All authors read and approved the final manuscript.

## Supplementary Material

Additional file 1 **Figure S1.** Specificity and efficiency of FGFR1 ablation in adipose tissue using the aP2 promoter. (A) Expression of the aP2 promoter in adipose tissue. The male reproductive complexes with attached gonadal adipose tissues in LacZ ROSA26R reporter mice (aP2Cre-) and the reporter mice crossed with aP2Cre mice (aP2Cre+) were analyzed by LacZ staining (blue). (B) Expression of the aP2 promoter in adipocytes. A paraffin-embedded section of the LacZ-stained (blue) gonadal fat tissue from (A) showed that more than 90 percent of adipocytes were positive for the aP2Cre recombinase activity. Tissue was counterstained with H&E. (C) FGFR1 mRNA expression among different tissues in FGFR1Fx and FGFR1Cn mice. FGFR1 expression was assessed by quantitative PCR. Total white adipose tissue (WAT) exhibited a 50% reduction of FGFR1 expression in the FGFR1Cn mice. *p<0.05 (n=5). (D) Relative expression of FGFR1, FGFR2 and KLB in mature adipocyte and the stromal-vascular (SV) fractions of adipose tissue. a: significant difference between adipocytes and sv fractions. b: significant difference between FGFR1Fx and FGFR1 Cn in the same adipocytes fraction or sv fraction. Data are the mean ± SD (n = 7-8), * p<0.05. Figure S2. Relative serum levels of FGF21 in the FGFR1Fx and FGFR1Cn mice at fed or fasted stages. The relative serum levels of FGF21 were measured by adipokine array kit (R&D systems) according to product manual. Sera were pooled from 3 mice (50 ul each mouse) for each genotype at fed state or after starved for 48 h. The dot blot membranes were analyzed for FGF21 antigen levels (A) and the relative intensity of spot was determined by densitometry (B). Figure S3. Lack of effect of the adipocyte FGFR1 deficiency on serum metabolic parameters in the fed state. Sera were collected from mice fed ad libitum within one hour after the start of the light part in the light–dark cycle. Imposed fasting for 4 h yielded similar results. The indicated parameters were assessed as described in text Figure 5. Data are the mean ± SD (n= 10), p<0.05 for all tests. Figure S4. Lack of effect of the adipocyte FGFR1 deficiency on metabolic gene expression in adipose tissue. Expression of the indicated genes as described in Additional file 1: Table S1 in FGFR1Fx and FGFR1Cn mice was assessed by quantitative PCR after 4 h fasting or 48 h starvation. The expression levels were standardized relative to those of FGFR1Fx mice with 4 h fasting, which were assigned a value of 1. Data are the mean ± SD (n= 10), p<0.05 for all tests. Figure S5. Effects of adipose FGFR1 deficiency on the expression of oxidative stress markers Ucp2 and Nrf2. mRNA levels for hepatic Ucp2 and Nrf2 were determined by quantitative PCR analyses. The expression level is relative to the FGFR1Fx under normal fed condition, which is considered as an arbitrary unit 1. Data are the mean ± SD of 6 mice for each group, p<0.05. Figure S6. Effects of adipose FGFR1 deficiency on serum enzyme activities for liver ALT and AST. Blood is collected from FGFR1Fx and FGFR1Cn mice after food starvation for 48 h, and serum is used to measure enzyme activities of liver-derived ALT and AST as a result of liver injury and diseases. Data are the mean ± SD of 6 mice for each group, * p<0.05. Table S1. Metabolic genes analyzed in expression analyses. Click here for file
