# Supplementary material for: Control of lipid metabolism by adipocyte FGFR1-mediated adipohepatic communication during hepatic stress
Source: Nutr Metab (Lond). 2012 Oct 30;9:94. doi: 10.1186/1743-7075-9-94 (PMC3545967; doi:10.1186/1743-7075-9-94)
Supplement: Additional file 1 — Figure S1. Specificity and efficiency of FGFR1 ablation in adipose tissue using the aP2 promoter. (A) Expression of the aP2 promoter in adipose tissue. The male reproductive complexes with attached gonadal adipose tissues in LacZ ROSA26R reporter mice (aP2Cre-) and the reporter mice crossed with aP2Cre mice (aP2Cre+) were analyzed by LacZ staining (blue). (B) Expression of the aP2 promoter in adipocytes. A paraffin-embedded section of the LacZ-stained (blue) gonadal fat tissue from (A) showed that more than 90 percent of adipocytes were positive for the aP2Cre recombinase activity. Tissue was counterstained with H&E. (C) FGFR1 mRNA expression among different tissues in FGFR1Fx and FGFR1Cn mice. FGFR1 expression was assessed by quantitative PCR. Total white adipose tissue (WAT) exhibited a 50% reduction of FGFR1 expression in the FGFR1Cn mice. *p<0.05 (n=5). (D) Relative expression of FGFR1, FGFR2 and KLB in mature adipocyte and the stromal-vascular (SV) fractions of adipose tissue. a: significant difference between adipocytes and sv fractions. b: significant difference between FGFR1Fx and FGFR1 Cn in the same adipocytes fraction or sv fraction. Data are the mean ± SD (n = 7-8), * p<0.05. Figure S2. Relative serum levels of FGF21 in the FGFR1Fx and FGFR1Cn mice at fed or fasted stages. The relative serum levels of FGF21 were measured by adipokine array kit (R&D systems) according to product manual. Sera were pooled from 3 mice (50 ul each mouse) for each genotype at fed state or after starved for 48 h. The dot blot membranes were analyzed for FGF21 antigen levels (A) and the relative intensity of spot was determined by densitometry (B). Figure S3. Lack of effect of the adipocyte FGFR1 deficiency on serum metabolic parameters in the fed state. Sera were collected from mice fed ad libitum within one hour after the start of the light part in the light–dark cycle. Imposed fasting for 4 h yielded similar results. The indicated parameters were assessed as described in text [file 1743-7075-9-94-S1.docx]

**SUPPORTING INFORMATION by Yang et al.**

Fig. S1. Specificity and efficiency of FGFR1 ablation in adipose tissue using the aP2 promoter. (A) Expression of the aP2 promoter in adipose tissue. The male reproductive complexes with attached gonadal adipose tissues in LacZ ROSA26R reporter mice (aP2Cre-) and the reporter mice crossed with aP2Cre mice (aP2Cre+) were analyzed by LacZ staining (blue). (B) Expression of the aP2 promoter in adipocytes. A paraffin-embedded section of the LacZ-stained (blue) gonadal fat tissue from (A) showed that more than 90 percent of adipocytes were positive for the aP2Cre recombinase activity. Tissue was counterstained with H&E. (C) FGFR1 mRNA expression among different tissues in FGFR1Fx and FGFR1Cn mice. FGFR1 expression was assessed by quantitative PCR. Total white adipose tissue (WAT) exhibited a 50% reduction of FGFR1 expression in the FGFR1Cn mice. *p<0.05 (n=5). (D) Relative expression of FGFR1, FGFR2 and KLB in mature adipocyte and the stromal-vascular (SV) fractions of adipose tissue. a: significant difference between adipocytes and sv fractions. b: significant difference between FGFR1Fx and FGFR1 Cn in the same adipocytes fraction or sv fraction. Data are the mean ± SD (n = 7-8), * p<0.05.


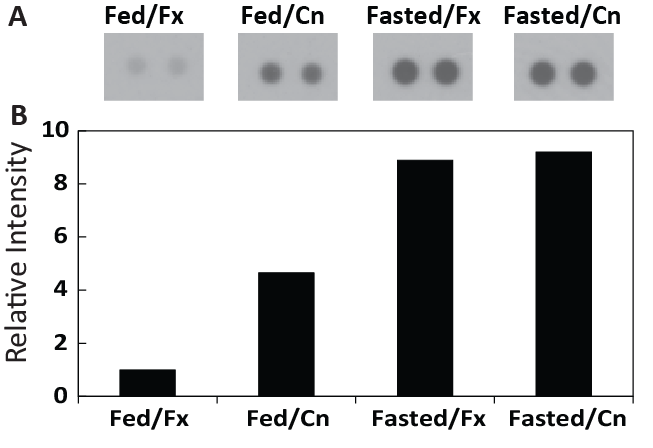


Fig. S2. Relative serum levels of FGF21 in the FGFR1Fx and FGFR1Cn mice at fed or fasted stages. The relative serum levels of FGF21 were measured by adipokine array kit (R&D systems) according to product manual. Sera were pooled from 3 mice (50 ul each mouse) for each genotype at fed state or after starved for 48 hrs. The dot blot membranes were analyzed for FGF21 antigen levels (A) and the relative intensity of spot was determined by densitometry (B).


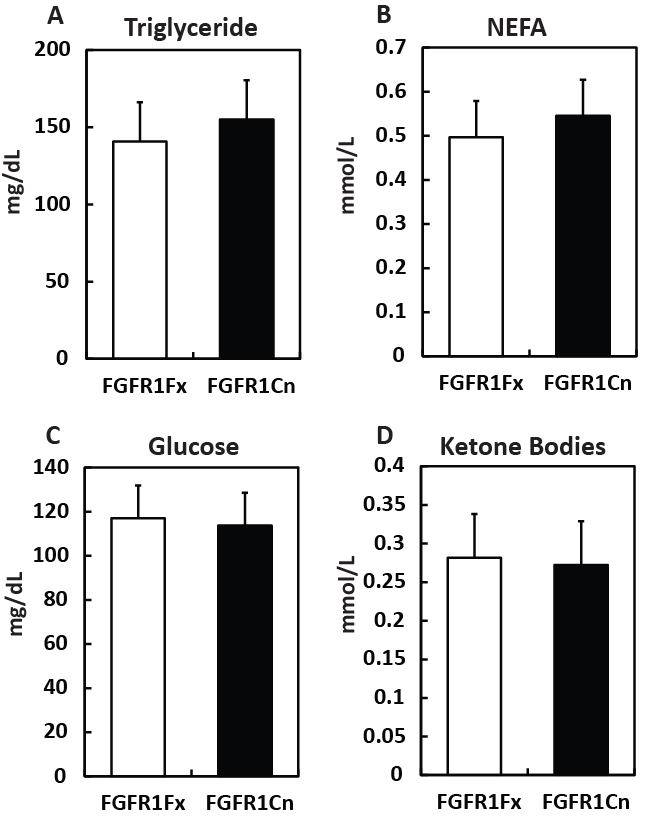


Fig. S3. Lack of effect of the adipocyte FGFR1 deficiency on serum metabolic parameters in the fed state. Sera were collected from mice fed *ad libitum* within one hour after the start of the light part in the light-dark cycle. Imposed fasting for 4 hrs yielded similar results. The indicated parameters were assessed as described in text Fig. 5. Data are the mean ± SD (n= 10), p<0.05 for all tests.


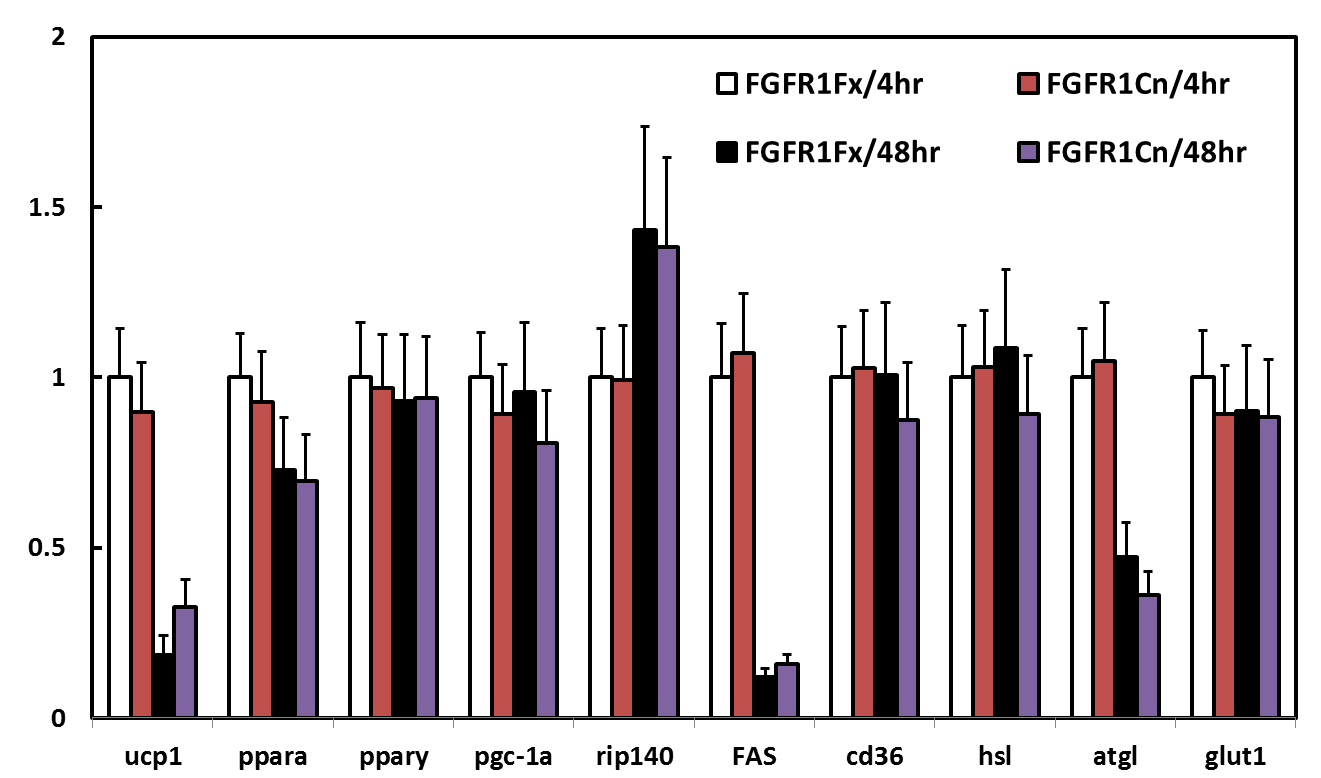


Fig. S4. Lack of effect of the adipocyte FGFR1 deficiency on metabolic gene expression in adipose tissue. Expression of the indicated genes as described in Table S1 in FGFR1Fx and FGFR1Cn mice was assessed by quantitative PCR after 4 hr fasting or 48 hr starvation. The expression levels were standardized relative to those of FGFR1Fx mice with 4 hr fasting, which were assigned a value of 1. Data are the mean ± SD (n= 10), p<0.05 for all tests.

Fig. S5. Effects of adipose FGFR1 deficiency on the expression of oxidative stress markers Ucp2 and Nrf2. mRNA levels for hepatic Ucp2 and Nrf2 were determined by quantitative PCR analyses. The expression level is relative to the FGFR1Fx under normal fed condition, which is considered as an arbitrary unit 1. Data are the mean + SD of 6 mice for each group, p<0.05.

 Fig. S6. Effects of adipose FGFR1 deficiency on serum enzyme activities for liver ALT and AST. Blood is collected from FGFR1Fx and FGFR1Cn mice after food starvation for 48 hr, and serum is used to measure enzyme activities of liver-derived ALT and AST as a result of liver injury and diseases. Data are the mean + SD of 6 mice for each group, * p<0.05.

**Table S1. Metabolic genes analyzed in expression analyses.**

| Lipogenic | CD36 | NM_007643 |
| --- | --- | --- |
|  | PPARγ | NM_011146 |
|  | LXR | NM_013839 |
|  | SREBP1C | NM_011480 |
|  | DGAT1 | NM_010046 |
|  | ACC1 | NM_133360 |
|  | ACC2 | NM_133904 |
|  | FAS | NM_007988 |
|  | SCD1 | NM_009127 |
| Lipolytic | AOX | NM_015729 |
|  | CPT1 | NM_013495 |
|  | MTP | NM_008642 |
|  | MCAD | NM_007382 |
| Others | PEPCK | NM_011044 |
|  | FXR | NM_009108 |
|  | SHP1 | NM_011850 |
|  | MnSOD | BC010548 |
|  | Nrf2 | BC026943 |
|  | Ucp2 | BC012697 |
| FGF21 Related | PPARα | NM_011144 |
|  | FGF21 | NM_020013 |
|  | PGC1α | NM_008904 |
| Adipose Related | UCP1 | NM_009463 |
|  | RIP140 | NM_173440 |
|  | HSL | NM_001039507 |
|  | ATGL | NM_001163689 |
|  | GLUT1 | NM_011400 |
| Standard | β actin | NM_007393 |
